# Methanolic extract of *Potentilla fulgens* root and its ethyl-acetate fraction delays the process of carcinogenesis in mice

**DOI:** 10.1038/s41598-019-53747-5

**Published:** 2019-11-18

**Authors:** Buddha Ganguly, Alka Chaudhary, Hughbert Dakhar, Inder Pal Singh, Anupam Chatterjee

**Affiliations:** 1 Molecular Genetics Laboratory, Department of Biotechnology & Bioinformatics, North-Eastern Hill University, Shillong, Meghalaya 793022 India; 2 Department of Natural Products, National Institute of Pharmaceutical Education and Research, Sector-67, S.A.S. Nagar, 160062 Punjab India; 3 Histopathology Division, Nazareth Hospital, Laitumkhrah, Shillong 793003 India

**Keywords:** Cancer prevention, Chromosome abnormality

## Abstract

People of north-eastern states of India consume raw areca-nut (RAN) and lime which could lead to oral, esophageal and gastric cancers. However, the incidence of these cancers are significantly lesser in those who consume pieces of *Potentilla fulgens* root along with RAN. Since evaluation of anticancer role, if any, of *P*. *fulgens* on RAN-mediated genetic alterations in human is difficult because of other compounding factors, this study was undertaken in mice to focus on gastric carcinogenesis since *ad libitum* administration of RAN extract with lime in drinking water induced stomach cancer due to greater exposure of its lining. A total of 160 mice were used at different time points and either methanol extract of *P*. *fulgens* roots (PRE) or mixture of four compounds of ethyl-acetate fraction (EA-mixture) was mixed with mice feed. Histological studies revealed that RAN + lime induced cancer in all the mice and interestingly only 20% developed cancer when PRE/EA-mixture was provided along with RAN + lime. Higher frequency of precocious anaphase and over expression of p53 and Securin genes were significantly reduced by PRE/EA-mixture. Thus PRE/EA-mixture mitigates the RAN-induced tumor-initiating process in stomach by maintaining expression of tumor suppressor and check-point genes under control.

## Introduction

Highest incidence of oral, esophageal and gastric cancers has been reported in the North-Eastern states of India^1^. Traditionally, people of these regions consume betel-quid which contains raw areca-nut (RAN), lime and a small part of betel-leaf but without tobacco. The whole betel-quid is swallowed after chewing and such a habit has been associated with development of these cancers^1–4^. A high frequency of gastric cancer in this region has also been associated with, besides RAN and lime, several causative factors like some non-vegeterian food items and alcohol. It has also been reported that *Helicobactor pylori* infection enhances development of gastric cancers caused by chewing and swallowing RAN and lime^5^. In animal model also, tumours have been shown to be induced in esophagous, stomach and liver when RAN extract was administered either by oral intubation^6^ or mixed with the diet^7^ or by ad libitum admisteration of RAN extract with lime in drinking water^8^. In earlier studies, induction of precocious anaphase (premature separation of sister-chromatids) and higher expression of p53 and Securin in non-target tissues like mouse bone marrow cells (BMC) were observed to be significantly associated with an increased risk of RAN-induced gastric cancer^8^. Subsequently, similar observation was made in human peripheral blood-lymphocytes of both oral and esophageal cancer patients and non-cancerous persons having the habit of consuming RAN^9^.

It is significant to note that some people in these regions who consume pieces of *Potentilla fulgens* roots along with the betel-quid show an extremely low incidence of these cancers^10,11^. *Potentilla fulgens* Wall. Ex Hook, commonly known as Himalayan Cinquefoil, is an important medicinal plant, because of its high ethnomedicinal value is well domesticated with the roots being commercially sold^12^. *Potentilla*, a member of Rosaceae family with about 500 species distributed across the world is nontoxic to human^13–15^. In Khasi Hills of Meghalaya, India. *P*. *fulgens* root-stock and whole herb is utilized as astringent and tonic for curing gum and tooth ailments, diarrhoea, diabetes and several other ailments^14–16^. The root extracts of *P*. *anserine* is used as a medicinal herbs against certain viral infection in Tibet^15–17^. *P*. *anserine* and other species have been used in traditional medicine in different parts of Europe and Northern America^18^. In China, different species of *Potentilla* have been used as folk medicinal herbs to treat diarrhoea, hepatitis and scabies^19^.

Natural products have been widely studied because their potential roles in prevention and treatment of cancer. By monitoring several well-studied markers and cellular processes for colorectal cancer it was demonstrated that natural product effectively mitigates the cancer development^20^. Significant anticancer activity has been reported for a number of triterpenoids compounds isolated from the aerial parts of *Potentilla chinensis* and the roots of *Potentilla multicaulis*^21^.

In view of the above noted observation that the incidence of cancer in persons exposed to RAN and lime together with roots of *P*. *fulgens* is significantly low, it is of interest to establish a causal mechanism. Since it is difficult to evaluate its anticancer role in RAN-mediated cancer in human system because of interference by other compounding factors like tobacco, alcohol consumption and diverse food-habits, many studies on anticancer effects of different *Potentilla* sp have been examined in human cancer cell lines^21–23^. The present study, we have examined the potential anticancer role of *P*. *fulgens* root extract (PRE) and its ethyl-acetate (EA) fraction on RAN associated gastric carcinogenesis in mice *in vivo*. Our present results confirm that PRE or four compounds of EA-fraction indeed have anticancer potentialities since their co-administration delays the RAN and lime induced gastric carcinogenesis.

## Results

### General observation

**Figure 1:**
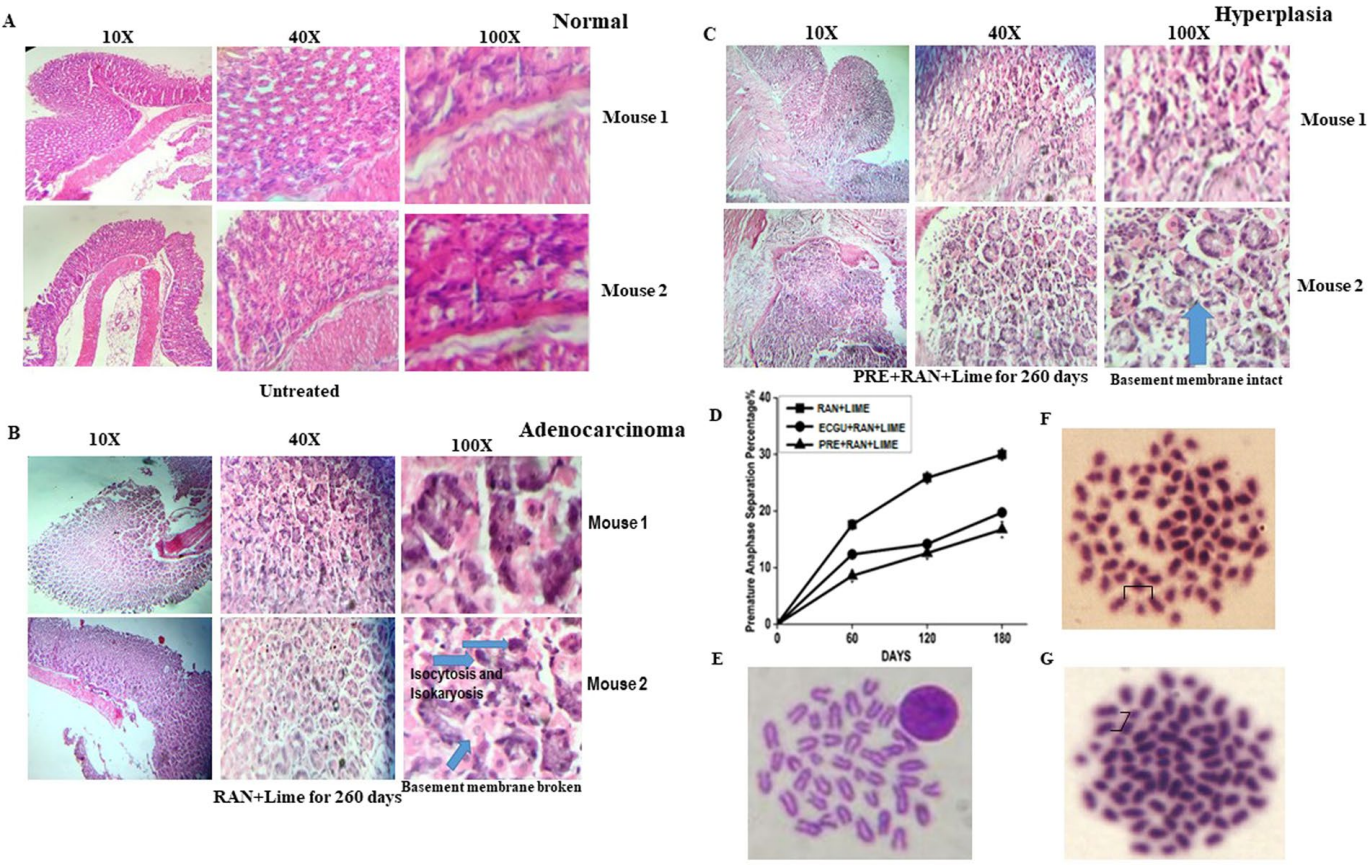
Histopathology of both normal and tumor tissues of stomach and karyotype analysis in bone marrow cells of mouse following treatment with RAN and lime with and without PRE for 260 days. (**A**) Mouse with normal stomach as untreated control. (**B**) Stomach with adenocarcinoma showing with isocytosis and isokaryosis (indicated with an arrow) in mouse fed with RAN + lime. (**C**) Histopathology of stomach with hyperplasia in mouse treated with PRE + RAN + lime. The magnification is indicated either 10X, 40X and 100X. (**D**) Percentage of metaphases with premature sister-chromatid separation. Five mice per point for untreated and 60 to 180 days of treatment with RAN + lime with and without PRE or ECGU (N = 4). At least 100 metaphases were scored to each mouse. (**E**) Normal metaphase spread from mouse bone marrow cells. (**F**,**G**) Premature sister-chromatid separation from mouse exposed to RAN + lime. Brackets show sister-chromatids lying separated in mitotic figures that show the phenotype.

In total, 160 mice were used at different time points for different experimental analysis (Table 1). Mice treated with RAN + lime with and without PRE for 260 days (n = 10 at each point) were used for both immunohistochemistry and histopathological evaluation. The histological section clearly differentiated among untreated normal (Fig. 1A), hyperplasia (Fig. 1B) and adeno-carcinoma (Fig. 1C) in stomach. The histological preparation of stomach tissue was made from all the mice in both the treated and untreated group (n = 5). All 10 mice treated with RAN + lime developed cancer in stomach. However, two out of 10 mice treated with PRE + RAN + lime showed carcinoma whereas 5 showed hyperplasia, a precancerous lesion in stomach. Interestingly, 3 mice in the PRE + RAN + lime group showed normal stomach. After 260 days of treatment, cancer was observed to be induced in all the ten mice treated with RAN + lime whereas only 20% mice developed cancer after treatment with PRE + RAN + lime.

**Table 1:** Number of mice involved per point for each study for the analysis of raw areca-nut mediated Carcinogenesis in mice and effect of PRE or ECGU on it.

Parameters/Approaches	Untreated	RAN+Lime				PRE+RAN+Lime				ECGU+RAN+Lime			Total Mice
		60d	120d	180d	260d	60d	120d	180d	260d	60d	120d	180d	
Metaphase preparation	5	5	5	5		5	5	5		4	4	4	47
Western Blot	5			5	5			5	5			4	29
qPCR	10			6	6			6	6				34
8-Oxo-dG ELISA	5			5	5			5	5				25
Immunohis tochemistry	5*				10*				10*				25
Histopathology	5*				10*				10*				
Total	30	5	5	21	26	5	5	21	26	4	4	8	160

*Commonly used for both; d- days; RAN- raw areca nut; PRE- *Potentilla* root extract; ECGU: Epicatechin, Catechin, Gallic acid and Ursolic acid.

### Studies on metaphase spreads

Continuous exposure to RAN extract with lime induced chromosomal instability which was evaluated in mouse BMC. We scored metaphase spreads 3 h after colchicine treatment and the data revealed a gradual but significant increase in the frequency of prematurely separated sister chromatids (Fig. 1D) in RAN + lime treated mouse compared to untreated one (Table 2). The frequency of premature anaphase separation (PAS) was 23.8% and 27.8% after 120 and 180 days of continuous administeration of RAN + lime, respectively. Interestingly, the frequency of PAS was reduced significantly if either PRE or mixture of epicatechin (E), catechin (C), gallic acid (G) and ursolic acid (U) (ECGU) was present along with RAN + lime (Fig. 1D, Tables 2 and S2 [Supplemetary section]). A normal metaphase cell showed 40 chromosomes (2 N = 40) in mouse BMC (Fig. 1E) whereas Fig. 1F,G showed metaphase spreads with prematurely separated sister chromatids. We performed chromosome count on metaphase spreads to test the importance of precocious anaphase on chromosome stability. The untreated mice showed a stable (2n = 40) karyotype without showing any precocious anaphase and aneuploid cells (Table 2). The frequency of aneuploidy cells was increased gradually from 0.7 to 2.6% after 60 to 180 days of treatment with RAN + lime, respectively. Interestingly, the frequency of both precocious anaphase and aneuploidy cells were reduced significantly when mice fed with either PRE (Table 2) or ECGU along with RAN + lime (Table S2).

**Table 2:** Chromosome analysis of mouse bone marrow cells after exposure to RAN extract with lime and PRE.

Treatment Pattern	Days of treatment	Total Plates scored	Chromosome No				PAS%	Mean±SEM	
			37	38	39	40		Aneuploidy%	PAS%
Untreated	0	115				115	0	0	0
		110				110	0		
		112				112	0		
		105				105	0		
		104				104	0		
RAN+L	60	107	2			105	13.1	1.0	15.7 ± 1.1
		110	1			109	16.4		
		100				110	19.0		
		105				105	16.2		
		104		1	1	102	13.7		
PRE+RAN+L	60	100				100	06.0	0.6	08.4 ± 0.8*
		110	1			109	08.1		p = 0.0006
		105		1		104	10.5		
		103				103	09.7		
		105			1	104	07.6		
RAN+L	120	110	1	1		109	26.0	1.8	23.8 ± 1.3
		106	1	2		103	27.0		
		116	1			115	21.6		
		109	1			108	23.9		
		112	2	1		109	20.4		
PRE+RAN+L	120	100	1			100	13.0	0.7	12.4 ± 0.8*
		105	1		1	103	14.6		p = 0.0001
		110				110	12.7		
		115				115	10.0		
		106				105	11.7		
RAN+L	180	106	1	2	1	102	28.3	2.6	28.0 ± 0.7
		101	1	1		099	28.7		
		115	2			113	27.8		
		110		2	1	107	25.5		
		105	2	1		102	29.5		
PRE+RAN+L	180	100				100	17.0	1.7	17.0 ± 1.6*
		107	1	2		104	14.9		p = 0.0003
		103	3			100	17.5		
		110	1			109	12.7		
		105	1	1		103	22.7		

*Significant in unpaired t-test between RAN + lime and PRE + RAN + lime.

p-values are shown and considered significant when the values are less than 0.05; Student’s t-test between RAN + lime and PRE + RAN + lime treated.

### PRE reduced oxidative DNA damage induced by RAN + lime

**Figure 2:**
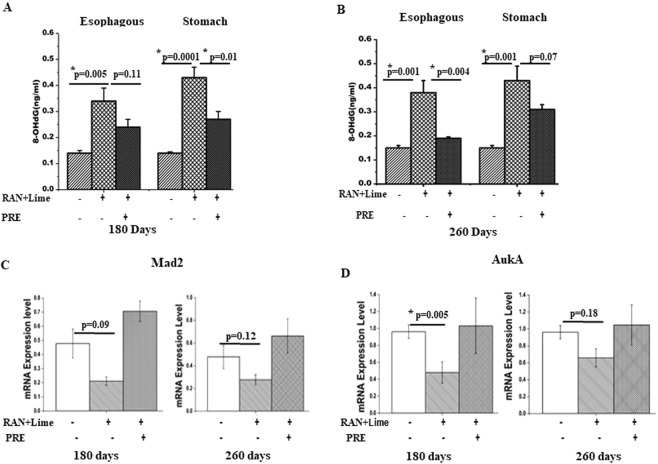
To see the effect of RAN + lime with and without PRE on 8-OHdG induction and the expression of mitotic regulator genes. (**A**) Quantitation of 8-OHdG (ng/ml) in DNA digests was performed by ELISA-kit in esophagous and stomach cells of mice treated for 180 days and (**B**) for 260 days. Data are plotted as a histogram. Each bar is the mean ± SEM derived from N = 5 samples used in each category. Both the tissues were used from the same mouse. (**C**) RNA extracted from stomach cells of a mouse treated for 180 and 260 days and further analyzed by qPCR for Mad2 and (**D**) AukA genes expression. Data are the mean ± SEM of a representative experiment performed in untreated (N = 10) and treated (N = 6 in each category). The values are normalized to respective GAPDH values. p-values are shown in all and considered significant when the values are less than 0.05, Student’s t-test.

An ELISA method was used to detect 8-OHdG as a marker of oxidative DNA damage in DNA extracted from cells that were collected from the inner layer of esophagous and stomach from untreated control, RAN + lime and PRE + RAN + lime treated mouse for 180 and 260 days (5 mice per point) (Fig. 2A,B). It was clear that the level of 8-OHdG was increased after RAN + lime exposure and the degree of induction was more in stomach than esophagous. Interestingly, when mice were consumed PRE and RAN + lime together the level of 8-OHdG was reduced.

### PRE maintained the expression status of mitotic checkpoint genes in RAN + lime fed mice

We examined the expression of Mad2 and AuKA genes in cells from the inner layer of stomach of mice untreated (n = 10) or administered RAN + lime with or without PRE for 180 and 260 days (n = 6 per point). The quantitative RT-PCR results (Fig. 2C,D) showed that the expression of Mad2 and AuKA genes was reduced considerably in RAN + lime treated mice with respect to untreated control. However, such reduction in the expression was not seen when PRE was present. In fact, presence of PRE maintained the expression of both these genes almost similar to untreated control level.

### PRE or ECGU maintained the level of p53 and Securin protein: Immunoblotting studies

**Figure 3:**
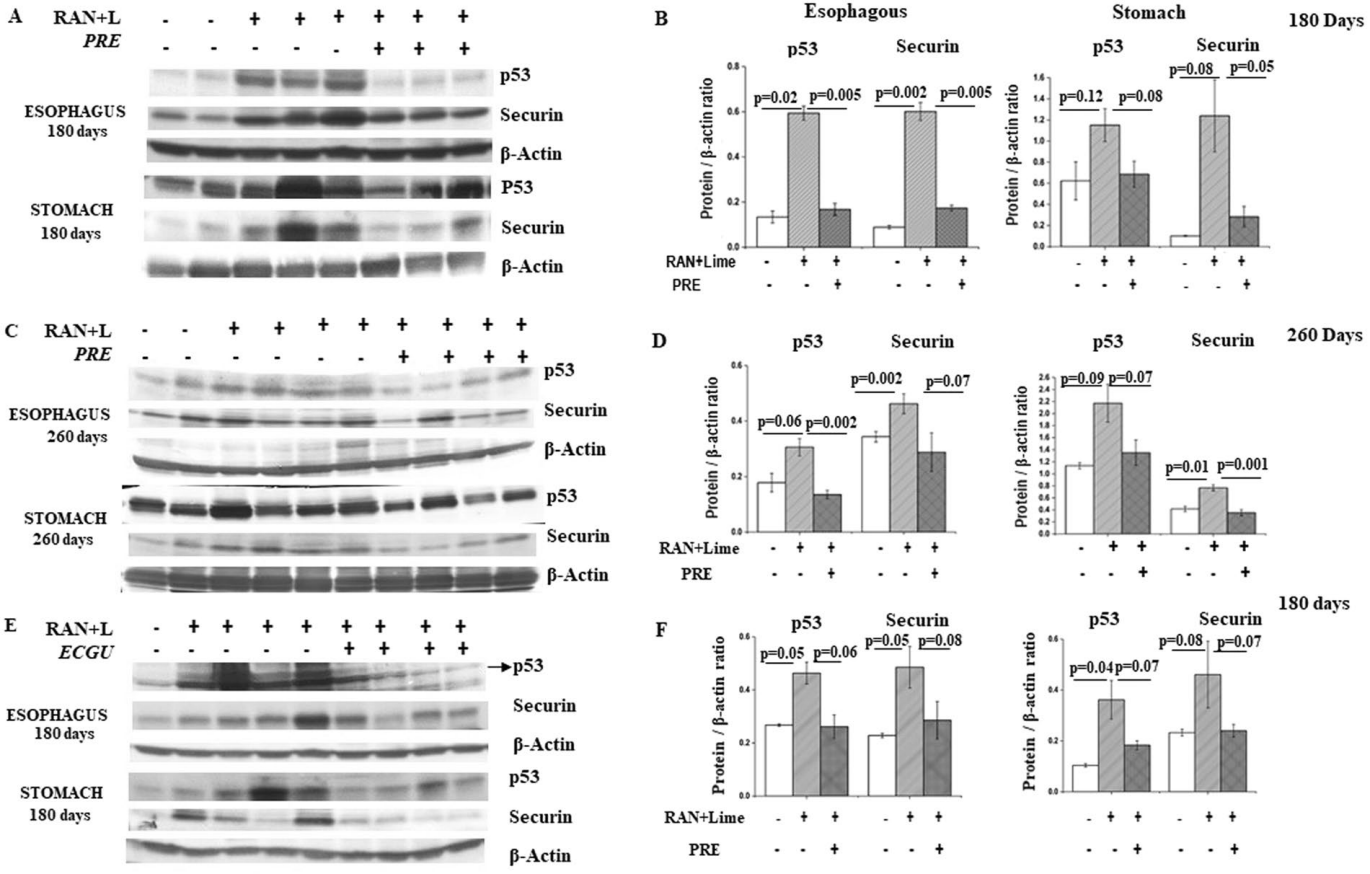
Representative western blotting detection of p53, Securin and β-actin in mouse esophagus and stomach cells after exposure with RAN + lime with and without PRE. (**A**) Two untreated control, 3 each treated with RAN + lime with and without PRE for 180 days. (**C**) Two untreated control, 4 each treated with RAN + lime with and without PRE for 260 days. (**E**) One untreated control, 4 each treated with RAN + lime with and without ECGU for 180 days. Arrow indicates the band. β-actin was used as loading control. Right side panel showed the quantitative densitometric analysis of the level of proteins of the above mentioned genes in (**B**) for the data showed in (**A**); in (**D**) for the data showed in (**C**) and in (**F**) for the data showed in (**E**). The values are the mean ± SEM of the number of individuals used in this experiment. The values are normalized to respective β-actin values. Both significant and non-significant p values are shown compared with negative/positive control (as determined by paired t-test). P-values less than 0.05 are considered significant.

Levels of p53 and Securin in cells from inner layer of esophagous and stomach of those mice which were untreated (n = 5) or administered RAN + lime with or without PRE for 180 and 260 days (n = 5 per point) were evaluated by immunoblotting. Similar analysis was also performed in mice administered RAN + lime with or without ECGU for 180 days. Results indicate that the level of these proteins were elevated significantly in both the tissues after administration of RAN + lime. However, such elevated level was reduced significantly in presence of either PRE or ECGU along with RAN + lime (Fig. 3A–F).

### Immunohistochemical staining for p53 and Securin in RAN induced cancer samples

**Figure 4:**
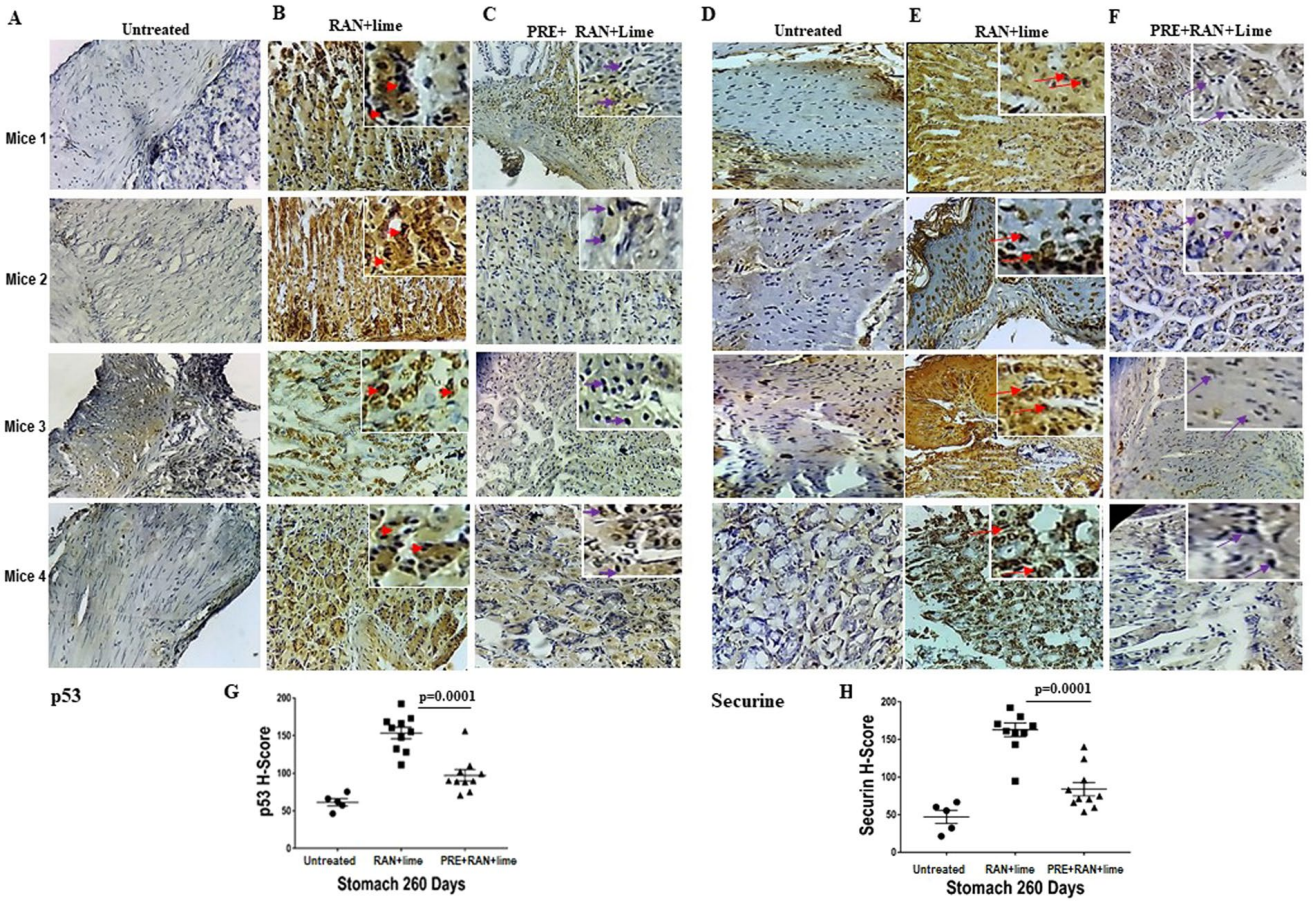
Representative images of an immunohistochemical (IHC) analysis of mouse stomach cells taken from untreated and treated with RAN + lime with and without PRE for 260 days. The normal expression of nuclear p53 (**A**) and nuclear Securin (**D**) genes in 4 untreated mice are shown. Overexpression of nuclear p53 (**B**) and both nuclear and cytoplasmic Securin (**E**) in RAN + lime induced cancer cells in 4 different mice are shown. The reduced expression of p53 (**C**) and securin (**F**) with respect to (**B**,**E**) are also depicted here in the 4 mice who were treated with PRE + RAN + Lime. The magnification of all these images is ×40. The enlarged inset in (**B**,**C**) show nuclear p53 and E shows both nuclear and cytoplasmic (arrowed inset in **E**: mice 2) and F shows nuclear Securin. (**G**) p53 expression levels and (**H**) Securin expression levels in untreated and treated with RAN + lime with and without PRE in mice analysed by H-score and were shown the mean H-score ± SEM. Data were analysed by Student’s *t*-test. Two-tailed *P* value was equal to 0.0001 between RAN + lime and PRE + RAN + lime treated.

We studied p53 and Securin expression by immunostaining in a panel of mouse stomach samples collected from untreated (n = 5) and treated with RAN + lime (n = 10) or PRE + RAN + lime (n = 10) for 260 days (Fig. 4A–F). The sections of stomach from RAN + lime treated (Fig. 4B,E) showed significantly higher expression of both p53 and Securin than samples collected from untreated mice (Fig. 4A,D). The expression of both p53 and Securin was observed in the nucleus but in cancer samples Securin was also seen in the cytoplasm as well (Fig. 4E, mice 2 and 4 in the enlarged inset with an arrow). This elevated expression of both p53 and Securin genes were reduced significantly when mice were treated with PRE + RAN + lime (Fig. 4C,F). H-score of p53 varied from 100 to 194 and for Securin it varied from 94 to 190 in RAN + lime treated samples (Fig. 4G,H). The mean H-score for p53 came down from 152 to 94 and for Securin it came down from 166 to 84 in PRE + RAN + lime treated mice.

## Discussion

Natives of north-eastern regions of India use *P*. *fulgens* root-stock and herb as folk-medicine against various ailments^16^. Evaluation of the total phenolic and flavonoid contents of *P*. *fulgens* root extract revealed the gallic acid and quercetin to be 138.8 ± 1.6 mg and 401.6 ± 4.6 mg per gm, respectively^22^. The root extract increased the survival of mice bearing Ehrlich ascites tumour cells and reduced cell viability in a dose-dependent manner in MCF-7 cells^22^. The ethyl-acetate (EA) soluble fraction of methanolic extract of *P*. *fulgens* root exhibited more efficient growth-inhibitory effect in MCF-7 and U87 cancer cell lines than hexane and n-butanol soluble fraction^23^. This observation prompted further purification of EA-fraction and a total of nine compounds including two new ursane type triterpenoids, Fulgic acid A and Fulgic acid B with a good antioxidant activity were identified^24^. Since the natural compounds act through diverse mechanisms and show minimal undesirable side effects^25^, it is attractive to evaluate anticancer activities of natural plant-based compounds in mammalian system.

In this study, mice were given the whole RAN-extract and lime *ad libitum* in drinking water with dose increased every two months to mimic the human habit of consumption of RAN. In earlier studies with similar mode of treatment, parameters like precocious anaphase and expression of Securin and p53 genes were monitored at different time-points starting from 30 days to 300 days for understanding the process of RAN-induced carcinogenesis^8^. It was found that after 240 days of *ad libitum* administration of RAN extract with lime in drinking water all the mice developed gastric tumour. Therefore, in the present study we monitored the influence of PRE on carcinogenesis after 180 and 260 days of treatment.

We examined the anticancer potency of PRE or of a mixture of epicatechin (E), catechin (C), gallic acid (G) and ursolic acid (U). in mice *in vivo*. These four compounds were selected primarily because of their higher yields: E (2.7%), C (1.6%), G (3.6%) and U (3.3%) in crude methanol extract^24^. Following the practice in *Potentilla* based traditional Chinese medicine^26^, we provided PRE or ECGU to experimental mice one month before RAN + lime treatment in order to strengthen their physiological system. In fact, it was suggested that such pretreatment of *Potentilla* based traditional Chinese medicine is fortifying the stomach, promoting the production of body fluid, invigorating the vital energy and nourishing the blood^26^.

Autoxidation of areca-nut polyphenols at alkaline pH and consequent generation of hydrogen peroxide and superoxide radicals accelerates RAN-induced carcinogenesis^2,27^. Further, transition metal ions such as Cu^−2+^, Mn^++^, Fe^2+^ and Fe^3+^ present in RAN and betel-leaf, facilitate the production of more reactive oxygen species which in turn contributes to initiation and promotion of cancer^28^. DNA is biologically significant target of oxidative attack, and it is widely believed that continuous oxidative damage to DNA is a significant contributor to the development of the major disorders including cancers. Among the diverse oxidative DNA damages, 8-hydroxy-deoxyguanosine (8-OHdG) is one of the most important DNA lesions and has been used as a marker for DNA oxidation^29^. Present results indicate that the high level of 8-OHdG following RAN + lime treatment was considerably reduced when PRE was co-administered. Thus, the presence of PRE or ECGU reduced DNA damages induced by RAN + lime mediated reactive oxygen species because both EA and butanol fractions of PRE showed a significant antioxidant potential, as evaluated by DPPH• and ABTS + • (hydrogen atom transfer based) and MTT assay system^24^. Moreover, Corosolic acid which is present in EA-fraction of PRE, is known to deplete the level of 8-OHdG and reduce the oxidative stress^24,30^.

Earlier studies highlighted the importance of precocious anaphase, which leads to aneuploidy, as a potential screening marker for identification of mitotic checkpoint defects during early days of RAN exposure in both mice and human^8,9,31^. As observed earlier^8^, present results also showed a gradual increase in the frequency of precocious anaphase and aneuploidy in the BMC of mice following RAN + lime administration. Very significantly frequencies of both were reduced following co-administeration of both PRE or ECGU. It is known that the percentage of aneuploid cells correlates with the severity of premature sister-chromatid separation, suggesting a direct relationship between the severity of loss of checkpoint control and chromosome mis-segregation^8,9,31^. Partial loss of spindle assembly checkpoint gene like MAD2 in Hct116 cells and also in mouse primary embryonic fibroblast cells showed increased premature sister chromatid separation in the presence of spindle inhibitors and higher rate of chromosome mis-segregation events in the absence of spindle inhibitors^31^. Another study, showed that arecoline, an alkaloid component of areca-nut, upregulated the spindle assembly checkpoint genes like Aurora A, BubR1 and Mps1 which led to mitotic spindles distortion and misalignment of chromosomes^32^. In this study, downregulation of MAD2 and AuKA in stomach cells of the mouse was noted after administeration with RAN + lime but in the presence of PRE, their expression was similar to untreated control. Thus, the inhibitory effect of RAN + lime on some of the spindle assembly checkpoint genes can be abrogated significantly by either PRE or ECGU.

Elevated expression of p53 protein is well documented in head and neck squamous cell carcinoma^33,34^ and also in oral dysplastic lesions and therefore such alteration in p53 is considered to be an early event in oral carcinogenesis^35^. Securin or pituitary tumor transforming gene, is known to be involved in the regulation of chromatid separation at metaphase-anaphase interface of the cell cycle^36^. Overexpression of Securin has been associated with the aneuploidy formation due to chromatid mis-segregation and is demonstrated in multiple cancer types^37,38^. Overexpression of p53 as well as Securin genes in non-target cells like mouse BMC and in human peripheral blood lymphocytes as well as in stomach and esophageal cells after exposure to RAN + lime has also been reported^8,30^. Interestingly, similar elevated expression of both these genes in stomach as well as in esophageal cells seen in this study after RAN + lime treatment was found to be considerably reduced by PRE or ECGU exposure. Since cancer induction mainly happened in stomach due to the greater exposure, further immunohistochemical analysis was performed only in stomach, which fully agreed with the immunoblotting data.

In agreement with the above, only 20% mice developed RAN + lime induced stomach cancer in presence of PRE whereas in its absence all the RAN + Lime exposed mice developed cancer. Thus the presence of PRE or ECGU delays the RAN + lime induced stomach carcinogenesis by maintaining the normal expression status of tumor suppressor and check-point genes. This seems to explain the greatly reduced incidence of oral and esophageal cancers in human when *P*. *fulgens* root is consumed with RAN + lime. It is interesting that the mixture of four compounds of EA-fraction showed similar potentiality in anticancer property as of the PRE. Both catechin and epicatechin are well known natural antioxidant and showed anticancer property^39,40^. Gallic acid can induce cell death of various cancer cells^41^ by depleting cellular glutathione and blocking EGFR signal pathway^42^. A pentacyclic triterpene ursolic acid has also been shown to have anti-inflammatory, antioxidant, and antitumor effects^43^. Consequently, all these compounds show anticancer potentialities with different targets.

This *in vivo* study thus demonstrated that the presence of either PRE or ECGU mitigates the RAN + lime induced tumor-initiating processes. Most strategies on drug development use either combination of several monotargeted drugs or a compound with multitargeting properties^20^. A recent, multiscale model for association of frequently mutated genes and inflammation mediated stomach, colon and liver cancer, based on information retrieved from the Gene Expression Omnibus, the Cancer Genome Atlas, and Gene Ontology database has suggested two stages of inflammation-induced tumorigenesis^44^. The present study, in agreement with earlier results^22,23^ indicated that PRE or ECGU targets precancerous state as well as the transition from the precancerous to tumorigenic states. Further in-depth studies are needed to identify the driver pathways targeted by PRE or ECGU in inflammation-induced tumorigenesis.

## Materials and Methods

### Plant material and extraction procedure

Roots of *Potentilla fulgens* were collected from ten different plants from Shillong peak regions of Meghalaya state of India (altitude 1700 m above sea level; 25°34 North latitude and 91°54 East longitude) after obtaining a proper approval of the forest officer. Taxonomic identification of the plant material was kindly confirmed by senior taxonomist of Department of Botany, North-Eastern Hill University, Shillong and a voucher specimen was deposited in the herbarium of the department (accession number 11906).

Extraction and isolation were performed earlier where ethyl acetate fraction was purified and all the compounds were identified and characterised^24,45^. Chemical structures were elucidated by spectroscopic methods, especially ESIHRMS and 2D NMR techniques^24,45^. In brief, *P*. *fulgens* roots (1 Kg) were shade dried and extracted with methanol using Soxhlet apparatus and obtained 250 gm of crude *Potentilla* root extract (PRE) which was suspended in water-methanol mixture (80:20) and partitioned with hexane, chloroform, ethyl acetate (EA), butanol and water soluble fractions. The EA-fraction was further subjected to vacuum liquid chromatography in silica-gel using hexane-ethyl acetate (0 to 90% EtOAc) and then chloroform-methanol gradients (1% to 50% MeOH) as elutent to yield five major fractions. It was already reported that nine compounds including new triterpenes and phenolics were identified and characterized from this EA-fraction of the methanolic root extract of *P*. *fulgens*^13^ (pl see the supplementary section for detailed isolation scheme Fig. S1). In this study, we used PRE or mixture of 50 µg each epicatechin (E), catechin (C), gallic acid (G) and ursolic acid (U) obtained from EA-fraction.

### Animals maintenance and treatment

Swiss albino mice weighing 25–30 gm and aged 2–3 months were maintained in community cages in a room with controlled temperature (20 ± 2 °C) and controlled lighting (12 h light; 12 h dark). Standard mouse diet (NMC Oil Mills Ltd., Pune, India) and water ad libitum were used in all experiments. The experiments were conducted in compliance with institutional guidelines and approved by North-Eastern Hill University “Institutional Ethics Committee (Animal Models)” Board.

In total, 160 mice were distributed into 4 groups: Group 1 was treated with simple drinking water considered to be untreated whereas group 2, 3 and 4 were administered RAN extract *ad libitum* in the drinking water with lime (pH 9.8) (Table 1). It was estimated that each mouse consumed 1 mg of extract per day till 60 days after which the dose was increased to 2 mg per day till 120 days. Likewise, every 60 days later the dose was increased by 1 mg per day consumption till it reached to 4 mg per day. In this study, mice were fed till 260 days.

In Group 3, PRE was given along with RAN + lime. In fact, PRE-treatment was started 30 days before RAN + lime treatment. PRE 2 mg powder was mixed with 6 gm of mice feed and finally made a tablet for each mouse. Thus, each mouse consumed 2 mg PRE every day. In group 4, epicatechin, catechin, gallic acid and ursolic acid mixture (ECGU) were used instead of PRE. A stock solution (5 mg/ml) of each compound was made and from this 10 µl of each was added to 6 gm of mice feed and finally made the tablet for each mouse. This way each mouse was consumed 50 µg of each compound every day.

### Chromosome preparation and scoring

For preparation of metaphase chromosomes, bone marrow cells (BMC) were collected 3 h after colchicine (15 mg/kg) treatment from 5 untreated mice as control. BMC were collected from untreated and 60, 120 and 180 days of different treated groups. The number of mice were used in each treated group are shown in Table 1. Animals were killed by cervical dislocation and femurs were dissected out for BMC which were treated with prewarmed KCl (0.075 M) and kept for 15 min at 37 °C. Then cells were fixed in acetic acid and methanol (1:3). Slides were prepared by flame drying method, stained with 5% Giemsa and mounted in a synthetic medium.

Around 100 well spread metaphase plates were studied for each mouse. We performed chromosome counts on metaphase spread. Values are expressed as mean ± SEMs.

### 8-OHdG measurement

Measurement of 8-Hydroxydeoxyguanosine (8-OHdG), a known marker of oxidative stress-mediated DNA damage, was estimated in DNA of esophageal and stomach cells with OxiSelect^™^ Oxidative DNA damage ELISA kit, Cell Biolabs Inc. (San Diego, CA) according to manufacturer’s instructions. Both esophageal and stomach tissues were collected from the mouse of untreated and treated with RAN + lime with and without PRE for 180 and 260 days. For 8-OHdG measurement, DNA were extracted and digested with nuclease P1 (Sigma, USA) and further treated with calf intestinal phosphatase (Sigma, USA) and denatured. 8-OHdG was quantified by quantitative ELISA assay in 96-well plate format. The quantity of 8-OHdG in the specimens were determined by comparing its absorbance with known 8-OHdG standard curve.

### RNA isolation and qRT-PCR analysis

Cells were collected from the inner layer of stomach from untreated mice (n = 10), RAN + lime and PRE + RAN + lime treated (n = 6 per point) for 180 and 260 days. Total RNA was extracted using an RNeasy Mini Kit (QIAGEN Co., Limburg, Netherlands). From 1 μg of total RNA, cDNA synthesis was performed using QuantiTect Reverse Transcription kit (Qiagen GmbH, Hilden, Germany) according to the manufacturer’s protocol. For qPCR, cDNA was amplified using SYBER Green PCR mastermix according to the manufacture’s cycling condition for 40 cycle on a StepOnePlus amplification and detection system (Applied Biosystems). The primers of target genes used for this analysis were Mitotic arrest deficient 2 (Mad2) and Aurora A Kinase (AukA), and the primer sequences are listed in Table S1 (Supplementary section). Data were analysed using the delta-delta Ct method and plotted as fold change versus control (pl see Supplementary section).

### Immunoblotting

Cells were collected from the inner layer of esophagous and stomach from untreated (n = 5), RAN + lime and PRE + RAN + lime treated mice for 180 and 260 (n = 5 per point) days. In case of 180 days treatment with ECGU + RAN + lime 4 mice were used as a treated group. The cells were washed with ice-cold 0.1 M phosphate-buffered saline (PBS; pH 7.4) and total protein was extracted with a lysis buffer containing 0.1% SDS, 2 mM EDTA, 1% NP-40, 1% sodium deoxycholate, 50 mM sodium fluoride, 100 U/ml aprotinin and 1 mM phenylmethylsulfonyl fluoride. After centrifugation, the cell lysate was collected and the protein concentration was determined using the bicinchoninic acid protein assay. Equal amount of protein (40 µg/well) were subjected to Novex Tris-Glycine 4–20% gradient gels and electrophoresis was performed in NuPAGE electrophoresis system (Invitrogen, USA). Then the proteins were transferred to a polyvinylidene difluoride membrane (Sigma) and probed with 1:1000 dilution of a mouse monoclonal antibody against p53 (PAb 240; ab-26; Abcam, USA), Securin (DCS-280; ab3305; Abcam, USA) and β-actin (AC-15; ab6276; Abcam, USA). Alkaline–phosphatase conjugated anti-mouse IgG (Abcam, USA) used as secondary antibodies and immunodetection was performed by treating the blot with the substrate solution of BCIP/NBT (Bangalore Genei, India).

### Histopathological evaluation

Stomach tissue was collected from untreated (n = 5), RAN + lime treated and PRE + RAN + lime treated mice (n = 10 at each point) at 260 days. Tissue sections (5–7 μm) were processed for histological sectioning as per standard protocol^46^ and stained with hematoxylin and eosin^47^. Sections were then observed under a light microscope and photographed (Carl Zeiss, Germany).

### Immunohistochemistry analysis

For immunohistochemistry (IHC) analysis, a small part of stomach tissue were collected from untreated and treated (both RAN + Lime with and without PRE) mice which were used for histopathological evaluation and kept in formalin. The tissues were dehydrated, paraffin embedded and sectioned with a microtome (Leica). Briefly, after blocking for endogenous peroxidase activity, the sections were incubated with anti-p53 (PAb 240; ab26; Abcam, USA) and anti-Securin (DCS-280; ab3305; Abcam, USA) primary antibody. IHC analysis was performed with a Strept-Avidin Biotin Kit (Dako). The scoring of immunohistochemical stains in each specimen was determined using a histological score (H)^48^ (please see Supplementary section).

### Statistics

The results are expressed as mean ± SEM for control and treated samples. Statistical analysis was performed by Student’s t-test with GraphPad Prism software 5.1. The values were considered statistically significant, if the p value was 0.05 or less.

## Supplementary information

Methanolic extract of Potentilla fulgens root and its ethyl-acetate fraction delays the process of carcinogenesis in mice

## Data Availability

The data that generated and supports the findings of this study will be available by the corresponding author upon request.
