# Supplementary material for: Methanolic extract of Potentilla fulgens root and its ethyl-acetate fraction delays the process of carcinogenesis in mice
Source: Sci Rep. 2019 Nov 18;9:16985. doi: 10.1038/s41598-019-53747-5 (PMC6861273; doi:10.1038/s41598-019-53747-5)
Supplement: Supplementary file 1 — Methanolic extract of Potentilla fulgens root and its ethyl-acetate fraction delays the process of carcinogenesis in mice [file 41598_2019_53747_MOESM1_ESM.docx]

**Methanolic extract of *Potentilla fulgens* root and its ethyl-acetate fraction delays the process of carcinogenesis in mice**

Buddha Ganguly, Alka Chaudhary, Hughbert Dakhar, Inder Pal Singh, Anupam Chatterjee

**Supplementary Information**

*Isolation and structure elucidation of compounds*

The ethyl acetate fraction was selected for further purification of pure compounds and for this it was subjected to vacuum liquid chromatography to yield five pooled fractions, E1 to E5. From fraction E1, ursolic acid, euscaphic acid and corosolic acid were received through column chromatography. From fraction E2, two stereoisomeric triterpene acids, fulgic acid A and fulgic acid B were separated by HPLC. Phenolics catechin, epicatechin, gallic acid, p-hydroxybenzoic acid, various monomeric and dimeric flavan-3-ols were obtained from fraction E3 to E5 after column chromatography and reverse phase HPLC. Chemical structures were elucidated by spectroscopic methods, especially ESIHRMS and 2D NMR techniques.

*
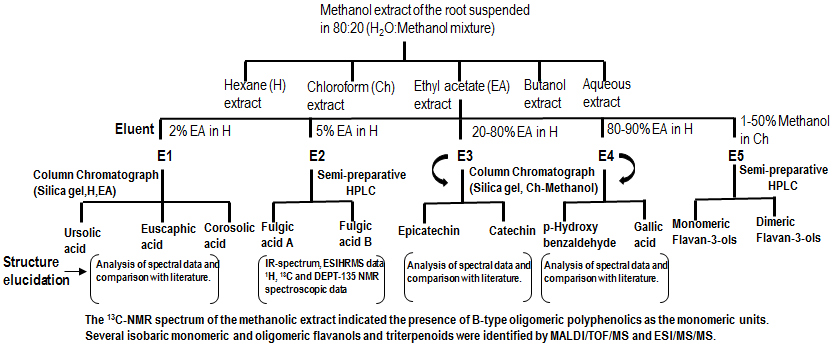
*

**Fig S1.** Isolation and characterization scheme scheme of *Potentilla fulgens* root extract

*Primer sequences*

Both forward and reverse primers of AuKA, Mad2 and GAPDH genes were used in qRT-PCR analysis. The sequences of the primers are mentioned below. These PCR primers for the specific genes were designed using primer design software Primer3 and verified for specificity using BLAST software.

**Table S1 Primer sequences**

AuKA (Forwad): ACATCCTCAGGCTGTATGGCTATTT

(Reverse): CCGTTTGAGCCAAGCAGTAAGTTCT

Mad2 (Forward): GCCGAGTTTTTCTCATTTGG

(Reverse): TTTGAGATGACCACCACCAG

GAPDH (Forward): ACAGTCCATGCCATCACTGCC

(Reverse): GCCTGCTTCACCACCTTCTTG

**RNA isolation and qRT-PCR analysis**

The qPCR was performed using 96-well optical reaction plates (Applied Biosystems, Darmstadt, Germany) using a StepOnePlus amplification and detection system (Applied Biosystems). The relative quantitation was done by using SYBR® Select Master Mix (Life Technologies), and the following conditions were used: 95°C for 5 min, 40 cycles of 95°C for 30 s, 60°C for 30s and 72^o^C for 30s. GAPDH was selected as the reference gene. The gene copy numbers of Mad2 and AukA were calculated by using a standard curve that was constructed using the OE33 cell line. The level of expression for the target gene was calculated as the ratio of the copy number of the target gene to that of the reference gene (GAPDH).

**Immunohistochemical analysis**

IHC staining was performed on formalin fixed, paraffin embedded tissues that were sectioned at 5 µm thickness. Slides were deparaffinized, rehydrated and treated with 3% H_2_O_2_ in PBS. Antigen retrieval was done with 0.01% sodium-citrate buffer followed by blocking in PBST containing 0.1% BSA and 10% FBS. Primary antibody incubation was carried out for overnight. Slides were washed and incubated with appropriate biotinylated secondary antibody for 1 hr at room temperature. Following washing slides were treated with streptavidin-HRP (1:1000) and subsequently washed (PBS containing 0.1% Tween-20) and color was developed with DAB+ H_2_O_2_. Slides were counterstained with haematoxylin, washed and mounted in DPX (Sigma-Aldrich, USA). The staining intensity of the images was evaluated by two independent investigators.

In the regions of the histologic sections, the chromogenic immunolabeling was systematically categorized into four groups: 0 (no labelling), 1+ (weak labelling), 2+ (moderate labelling), and 3+ (strong staining; observable with 10x objective). Semi-quantitative staining analysis was done by H-score by counting 400 cells from five different fields in the slides considering low, moderate and higher intensity of p53 and Securine expression. The percentage of positive cells with a given intensity for each sample was determined independently by a pathologist and a trained reader. A single manual H-score based on a scale of 0 to 250 was generated for each labelled section by taking the sum of the percentage of cells labelling 1+, double the percentage of cells labelling 2+, and triple the percentage of cells labelling 3+ (H-Score = ((%3+) × 3) + ((%2+) × 2) + (%1+)). Student’s t-test was performed for comparing the expression of these two genes in untreated samples with the samples treated with RAN+lime or RAN+lime+PRE. Statistical significance was considered when p-value is less than 0.05.

**Table S2**. **Chromosome analysis of mouse bone marrow cells after exposure to RAN extract with lime and ECGU**

Treatment Treatment Total Spread PAS % PAS % Mean

Pattern Days Score ± SEM ± SEM

______________________________________________________________________________

RAN+L 60 110 10.9 12.4 ± 0.5*

+ECGU 105 12.5 p=0.028

107 13.2

100 13.0

RAN+L 120 110 13.6 14.2 ± 0.7*

+ ECGU 100 13.0 p=0.0004

108 13.9

105 16.2

RAN+L 180 100 20.0 19.8 ± 0.8*

+ ECGU 107 20.6 p=0.0001

103 17.5

110 20.9

L=lime; PAS= premature anaphase separation; * statistically significant in paired t-test; two-tailed p value was shown. Each one shows a significant difference with the treatment of RAN+lime only in the matching treatment days. The RAN+lime data are shown in Table 2.
